# Differential binding of tropomyosin isoforms to actin modified with *m*-maleimidobenzoyl*-N-*hydroxysuccinimide ester and fluorescein-5-isothiocyanate

**DOI:** 10.1016/j.ab.2009.07.004

**Published:** 2009-11-01

**Authors:** Radosław Skórzewski, Katarzyna Robaszkiewicz, Justyna Jarzębińska, Piotr Suder, Jerzy Silberring, Joanna Moraczewska

**Affiliations:** a Institute of Experimental Biology, Kazimierz Wielki University in Bydgoszcz, 85-064 Bydgoszcz, Poland; b Faculty of Chemistry, Jagiellonian University, 30-060 Kraków, Poland

**Keywords:** Maleimidobenzoyl–actin, Cross-linked actin, FITC–actin, Mass spectrometry, Tropomyosin isoforms

## Abstract

Differential interactions of tropomyosin (TM) isoforms with actin can be important for determination of the thin filament functions. A mechanism of tropomyosin binding to actin was studied by comparing interactions of five αTM isoforms with actin modified with *m*-maleimidobenzoyl-*N*-hydroxysuccinimide ester (MBS) and with fluorescein-5-isothiocyanate (FITC). MBS attachment sites were revealed with mass spectrometry methods. We found that the predominant actin fraction was cross-linked by MBS within subdomain 3. A smaller fraction of the modified actin was cross-linked within subdomain 2 and between subdomains 2 and 1. Moreover, investigated actins carried single labels in subdomains 1, 2, and 3. Such extensive modification caused a large decrease in actin affinity for skeletal and smooth muscle tropomyosins, nonmuscle TM2, and chimeric TM1b9a. In contrast, binding of nonmuscle isoform TM5a was less affected. Isoform’s affinity for actin modified in subdomain 2 by binding of FITC to Lys61 was intermediate between the affinity for native actin and MBS-modified actin except for TM5a, which bound to FITC–actin with similar affinity as to actin modified with MBS. The analysis of binding curves according to the McGhee–von Hippel model revealed that binding to an isolated site, as well as cooperativity of binding to a contiguous site, was affected by both actin modifications in a TM isoform-specific manner.

Interactions of actin filaments with actin-binding proteins and myosin motors play a central role in muscle contraction and in many functions of nonmuscle cells [1]. Tropomyosin (TM) 1 is a thin filament regulatory protein that controls actin interactions with other proteins. Approximately 40 TM isoforms are expressed in tissue-dependent and developmentally dependent manners. All TM isoforms are α-helical coiled coils that bind head-to-tail to form continuous chains along the actin filament. Mammalian high-molecular-weight (HMW) isoforms extend along seven actin monomers, whereas low-molecular-weight (LMW) isoforms bind to six actin monomers. TM isoforms bind to actin with various affinities and show different abilities to regulate the actin filament [2,3]. These differences among TM isoforms are thought to be important factors in the diversification of actin filaments’ functions in various cell types [3]. Therefore, precise assembly of actin–TM complexes can be crucial for proper functioning of the actin filaments.

TM binding to actin is a highly cooperative process. This means that a TM molecule that is already attached to actin enhances the binding of a contiguous TM molecule. Cooperativity facilitates full saturation of the filament with TM because random binding, which could form gaps too short to be filled by one TM, is unfavorable. Because several factors can affect TM–actin interactions, the mechanism of cooperativity is still a matter of debate. Direct association of N and C termini of the adjacent TMs seems to be the primary source of cooperativity; however, many data suggest that allosteric conformational changes in actin are equally important (for a recent review, see Ref. [4]). Therefore, mapping the sites of TM–actin interactions and conformational changes within the actin scaffold associated with TM binding is important for our understanding of the mechanism of thin filament assembly.

In several investigations, the above problem has been approached by analysis of TM binding to actin modified by limited proteolysis [5–7], mutations [8–10], and attachment of chemical or fluorescent probes [11,12]. Actin modified with the heterobifunctional reagent MBS (*m*-maleimidobenzoyl-*N*-hydroxysuccinimide ester) has proven to be useful in studies of actin–myosin regulation by tropomyosin/troponin complex [13,14]. However, interpretation of the results in terms of actin structural changes is speculative because the localization of modified residues is not known.

In this work, we analyzed interactions of five muscle and nonmuscle TM isoforms with actin chemically modified by attachment of MBS (MB–actin). To be able to interpret the data, we identified the maleimidobenzoyl attachment sites in MBS-labeled actin using mass spectrometry (MS) methods. We found that subdomain 3 is the major site of modification but that subdomains 2 and 1 also carry MBS adducts. Comparative analysis of the affinities of TM isoforms to MB–actin and to fluorescein-5-isothiocyanate (FITC)–actin (actin derivative modified in subdomain 2 by attachment of FITC to Lys61) allowed us to demonstrate that actin subdomains 2 and 3 differentially participate in interactions with different TM isoforms. Isoform-specific sequences of TMs determine preferred sites of TM binding to the actin as well as cooperativity of the filament assembly.

## Materials and methods

### Isolation of actin and α-tropomyosin isoforms

G-actin was prepared from chicken skeletal muscle according to the method of Spudich and Watt [15]. After isolation, the protein was stored in G-buffer (2 mM Hepes [pH 7.6], 0.2 mM adenosine triphosphate [ATP], 0.1 mM CaCl_2_, and 0.01% NaN_3_) and was used within a week. Actin concentration was determined spectrophotometrically from absorbance at 290 nm using an extinction coefficient of 0.63 mg ml^−1^ cm^−1^ for 0.1% actin (MW 42,000).

Nonmuscle tropomyosin isoforms TM2, TM5a, and smooth muscle TM (smTM) used in this study are products of the rat αTM gene (TPM1). TM1b9a is a chimeric protein that is identical to TM5a except for the C-terminal 27 amino acids that are encoded by striated muscle-specific exon 9a [16]. The proteins were expressed in the *Escherichia coli* strain BL21 and then purified as described in Ref. [7]. Skeletal muscle αTM (skTM) was obtained from chicken pectoral muscle according to the method described in Ref. [17]. Rat and chicken skeletal αTMs share 96% identity and 97.5% sequence homology (NCBI Entrez Protein accession numbers AAA21801 and AAA48610). Unlike recombinant proteins produced in bacteria, skTM is N-terminally acetylated. TM2, smTM, and skTM are HMW tropomyosin isoforms. TM5a and TM1b9a belong to the LMW class of TM isoforms. In HMW isoforms N-terminal amino acids are encoded by exons 1a and 2, whereas in TM5a and TM1b9a this sequence is replaced by exon 1b. The sequence encoded by exon 2 (residues 39–80) was either smooth muscle-specific 2a or constitutive 2b. The C-terminal sequences of the isoforms used are encoded either by striated muscle-specific exon 9a or constitutive exon 9d found in smooth muscle and nonmuscle isoforms [18].

### Actin modification with MBS

Actin modification with MBS was performed as described by Bettache and coworkers [19] with modifications. G-actin concentration was kept between 2.8 and 3.8 mg/ml. MBS (25 mM in dimethyl formamide (DMF)) was slowly added under constant stirring to achieve 20-molar excess of the reagent over G-actin. The labeling was carried out for 16 to 20 h on ice. Unlabeled actin was polymerized in the presence of 0.1 M KCl and 2 mM MgCl_2_. After 1 h incubation at room temperature, F-actin was removed by 1.5 h ultracentrifugation at 45,000 rpm and 4 °C in a Beckman 70Ti rotor. The supernatant containing MBS-labeled actin was collected and dialyzed overnight against G-buffer. After dialysis, MB–actin was ultracentrifuged as above and the supernatant was filtered through Millipore 0.2-μm filters. The protein concentration was determined densitometrically based on the intensities of protein bands separated on 12% sodium dodecyl sulfate–polyacrylamide gel electrophoresis (SDS–PAGE) and stained with Coomassie blue. Known concentrations of native actin were used for preparation of a standard curve. Intensities of protein bands were measured using the software EasyDens (Cortex Nova, Bydgoszcz, Poland). The labeled actin was polymerized overnight in the presence of 0.1 M KCl, 2 mM MgCl_2_, and 1.5-molar excess of phalloidin to obtain MB–actin filaments.

### Actin modification with FITC

Labeling of actin with FITC was carried out according to the method of Burtnick [20] with slight modifications. G-actin was incubated with a 30-molar excess of FITC for 20 to 24 h on ice in G-buffer (0.2 mM ATP, 0.1 mM CaCl_2_, and 2 mM Hepes, pH 8.6). Unlabeled actin was removed by polymerization in the presence of 0.1 M NaCl and 2 mM MgCl_2_, followed by ultracentrifugation for 2 h at 45,000 rpm in a Beckman 70Ti rotor. To remove unbound FITC from FITC–actin preparation, the supernatant was gel-filtered through the Sephadex G-100 column and the collected fractions were concentrated using a Millipore Amicon Ultra-15 Ultracell 30K concentrator. Labeled protein was dialyzed against G-buffer (pH 7.6). FITC–actin was ultracentrifuged prior to use to remove denatured protein. Protein concentration was determined densitometrically as described above. The FITC concentration was determined spectrophotometrically at 460 nm using an absorption coefficient of 74,500 M^−1^ cm^−1^. The labeling ratio was determined to be 1.03 ± 0.06. Filamentous FITC–actin was obtained by polymerization with 0.1 M KCl, 2 mM MgCl_2_, and 1.5-molar excess of phalloidin.

### Chemical determination of thiol, amine, and maleimide groups

Reactive thiol groups were measured with Ellman’s colorimetric assay [21] using 5,5′-dithio-bis(2-nitrobenzoic acid) (DTNB) absorbance at 412 nm. *N*-Acetyl-l-cysteine was used for a standard curve. Actin unfolding was performed by incubation for 6 min with 5% SDS at 56 °C. Fully folded actin, as well as unfolded actin, was analyzed.

The total amount of lysine ε-amine was measured after protein denaturation by incubation for 6 min with 5% SDS at 56 °C. The analysis was done with the use of 2,4,6-trinitrobenzenesulfonic acid (TNBS), as described in Ref. [22], except that pH was controlled by Na_2_B_4_O_7_ (pH 9.5) and TNBS concentration was 0.005%. The number of amine groups was calculated using a molar extinction coefficient of 11,650 M^−1^ cm^−1^ at 335 nm [19].

The presence of free maleimide groups in the MBS-modified actin was determined as described by Liu and coworkers [23] using consecutive reactions of β-mercaptoethanol and DTNB. The quantity of β-mercaptoethanol bound to maleimide groups was determined spectrophotometrically at 412 nm using a standard curve. Before the measurements, MB–actin was purified by filtration on a Sephacryl S-200 HR column to ensure that all traces of unbound MBS were removed.

### Protein analysis by MS

Nanospray ionization MS was performed on the Applied Biosystems QSTAR XL tandem MS (MS/MS) under the following instrumental conditions: declustering potential (DP) was set to 60 V, and spray voltage applied to the needle (ion source (IS)) was adjusted to 0.9 to 1.5 kV. Spectra were deconvoluted using the Bioanalyst software package. Before analysis, MB–actin and nonmodified actin were dialyzed against water and lyophilized. Native actin was dissolved in a 3:1:0.1 (v/v/v) mixture of water, methanol, and formic acid. The best solubility of MBS-modified actin was achieved in a 1:3 (v/v) mixture of formic acid and water. The analysis was accomplished in the Pomeranian Science and Technology Park Bio-Lab Centre (Gdynia, Poland).

For generation of tryptic peptide maps, the sample was separated on the 10% SDS–PAGE gels and stained with Coomassie blue. The washing of gel pieces, in-gel trypsin digestion, and peptide elution were performed as described previously [24].

Liquid chromatography–electrospray ionization–MS/MS (LC–ESI–MS/MS) analyses were performed using an Esquire 3000 instrument (Bruker Daltonics, Germany) with a quadrupole ion trap analyzer and a homemade nanospray ion source. Chromatographic separations were performed with the aid of an Ultimate nanochromatography system (LC Packings/Dionex, Netherlands) [24].

Peptide sequences were identified using the Mascot engine (Matrix Science, London, UK). The sequence of chicken skeletal muscle α-actin was obtained from the Swiss–Prot database (accession number P68139). Monoisotopic theoretical masses of actin peptides generated by trypsin digestion were calculated using PeptideMass software [25].

### Molecular distance calculation

The distance separating Lys and Cys residues in actin atomic structure was calculated by the PyMOL 0.99rc6 Open Source (DeLano Scientific, Palo Alto, CA, USA) using skeletal actin (PDB accession number 1j6z).

### Analysis of tropomyosin binding to actin

Tropomyosin binding to actin was studied by cosedimentation assay as described in Ref. [7] except that the concentration of NaCl in the buffer used for the analysis of HMW TM isoform binding was reduced from 0.15 to 0.10 M.

The apparent association constant (*K*_app_) of TM to actin was calculated by fitting the experimental data to the Hill equation:

(1) $$v=n{[\text{TM}]}^{\alpha \text{H}}K_{\text{app}}/1+{[\text{TM}]}^{\alpha \text{H}}K_{\text{app}}^{\alpha \text{H}}\text{,}$$

where *v* is fraction maximal TM binding to actin, *n* is maximal TM bound, [TM] is free [TM], and α^H^ is the cooperativity coefficient.

The equilibrium constant for the association of TM with an isolated binding site (*K*_0_) and the equilibrium constant for moving TM from an isolated binding site to a singly contiguous binding site (ω) were obtained by fitting the data to the McGhee–von Hippel equation:

(2) $$\frac{\upsilon }{c}=K_{o}(1-n\cdot \upsilon ){\left(\frac{(2\omega -1)(1-n\cdot \upsilon )+\upsilon -R}{2(\omega -1)(1-n\cdot \upsilon )}\right)}^{n-1}\times \left(\frac{1-(n+1)\cdot \upsilon +R}{2(1-n\cdot \upsilon )}\right)\text{;}$$

where *R* = {[1 − (*n* + 1)υ]^2^ + 4ωυ(1 − *n*υ)}^1/2^, υ is the number of moles of TM bound per mole of actin, *n* is the stoichiometry of TM binding to actin, and *c* is the concentration of free TM. Nonlinear fitting was done in SigmaPlot (Systat Software, San Jose, CA, USA).

## Results

### Chemical and electrophoretic analysis of MBS-modified actin

MBS as a bifunctional reagent can react with a protein’s amine and thiol groups, potentially leading to internal cross-links and single-site modifications. Because MB–actin was applied previously to investigations of actin regulation by skeletal TM [13,14], we used it to study mechanisms of interactions between actin and different TM isoforms.

The protocol for actin modification with MBS differed slightly from the procedure used by Bettache and coworkers [19,26]. To protect the native structure of actin, the reaction was carried out at 0 °C for a prolonged time (overnight) rather than at 20 °C for 2 h. In addition, we did not quench the reaction with dithioerythritol (DTE) and glycine, which we observed denatures actin. Despite the milder labeling conditions, the modification rendered actin unable to polymerize at physiological salt concentrations (0.1 M KCl and 2 mM MgCl_2_), as with Bettache and coworkers’ original procedure [19]. The addition of phalloidin restored the ability of MB–actin to polymerize, as reported previously [13].

The extent of actin modification with MBS was evaluated by determination of free amine and thiol groups. Cysteine thiol groups were determined in a fully folded actin as well as in unfolded protein in the presence of SDS at high temperature. The only cysteine residue reactive in native actin (Cys374) was efficiently modified in MB–actin. The results obtained with unfolded actin show that besides Cys374, one more Cys residue was blocked by MBS (Table 1 ).

The free amino groups were determined after actin thermal denaturation in the presence of SDS. We were able to detect only 15 residues, in contrast to Bettache and coworkers [19], who determined all 19 Lys residues present in native actin. This could be caused by a partial obstruction of the reaction with TNBS by binding of SDS to some ε-amine groups of lysine [22]. Therefore, to calculate the number of Lys residues modified with MBS, we subtracted the reactive amine groups determined in MB–actin from the groups determined in native actin. The results summarized in Table 1 indicate that approximately four Lys residues were modified by MBS.

**Fig. 1:**
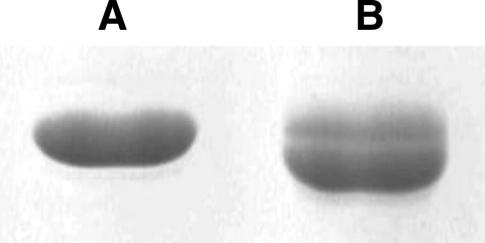
Electrophoretic analysis of MB–actin. Shown are native actin (A) and MBS-labeled actin (B) separated on 12% SDS–PAGE and stained in Coomassie blue.

MB–actin migrated as doublet bands in the SDS–PAGE (Fig. 1 ). The electrophoretic mobility of the upper band was the same as that of native actin, whereas the lower band migrated slightly faster. The densitometric ratio of upper to lower band intensities was 1:2.92 ± 0.11. Protein bands of higher molecular weights were not observed, indicating that the reaction with MBS did not produce intermolecular actin cross-links. The presence of doublet bands indicates that protein preparation is heterogeneous and contains a fraction of intramolecularly cross-linked actin [27].

### Characterization of MBS-modified actin with use of nanospray MS

To determine the stoichiometry of MBS adduct in differentially labeled actin species, the whole preparation of MB–actin was analyzed using nanospray MS.

**Fig. 2:**
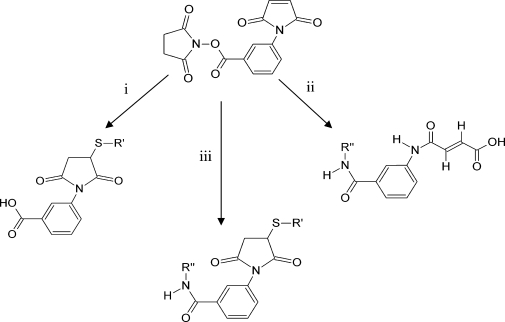
Reactions of MBS with protein side chains (based on Ref. [28]). Shown are binding of maleimidobenzoyl moiety to cysteine (i), *N*-hydroxysuccinimide reaction with lysine (ii), and cross-linking of lysine and/or cysteine residues (iii).

Theoretically, three types of modifications can be formed during reactions of MBS with a protein: (i) binding of the label to Cys side chain, (ii) binding to Lys side chain, and (iii) cross-linking of Lys to Cys/Lys side chains (Fig. 2 ). Binding of MBS to Cys SH group leads to maleimidobenzoic acid formation, causing an increase in protein molecular weight by 218 Da (Fig. 2, reaction i). Because actin’s N terminus is acetylated [29], the α-amine group on the N terminus is inaccessible and the reagent binds only to ε-amine groups of lysine side chains. Attachment of MBS to a Lys residue increases protein’s molecular weight by only 218 Da (Fig. 2, reaction ii). If a cysteine or another lysine residue falls within a distance of the maleimidobenzoyl spacer arm (9.9 Å), a Lys-Cys or Lys-Lys cross-link can be formed (Fig. 2, reaction iii). The cross-linked protein’s molecular weight is then increased by 199 Da. An indication that such a sequence of reactions takes place during the actin modification process is the absence of free maleimide groups in our MB–actin preparation, as verified by the reaction with β-mercaptoethanol (see Materials and Methods).

**Fig. 3:**
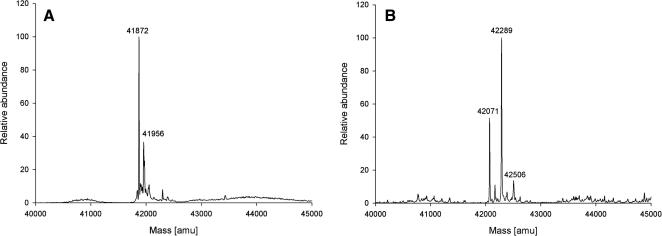
Deconvoluted nanospray MS spectra of native actin (A) and MB–actin (B).

Fig. 3 shows representative deconvoluted nanospray MS spectra of unmodified actin (Fig. 3A) and MB–actin (Fig. 3B). The native actin shows signal at a molecular weight of 41,872 ± 2 Da, in excellent agreement with the theoretical molecular weight calculated using PeptideMass for skeletal N-acetylated α-actin methylated at His73 residue (41,872 Da) [29]. A less abundant peak at a molecular weight of 41,956 was always observed in spectra of native actin. This signal may represent actin fraction modified by two azide ions from the antibacterial agent sodium azide. The peak characteristic for native actin was not observed in the MB–actin spectrum, showing that all actin molecules in the preparation were modified by MBS.

MBS modification gives rise to three major actin derivatives with molecular weights of 42,071 Da (I), 42,289 Da (II), and 42,506 Da (III). As compared with native actin, the molecular weight of derivative I is shifted by 199 Da. This additional mass corresponds to the maleimidobenzoyl moiety attached to the protein during cross-link formation, indicating that derivative I contains single internal cross-link. A mass increase by 417 Da in the molecular weight of derivative II accounts for the presence of two adducts. One of the adducts (199 Da) is the cross-link, and the second one (218 Da) is MB moiety bound to one Lys or Cys residue. The extra mass (634 Da) present in derivative III can be ascribed to one cross-linked group (199 Da) and two un-cross-linked groups (2 × 218 Da – 1 H^+^) attached to actin molecule.

### Determination of MBS-modified sites of actin by LC–ESI–MS/MS

To identify the sites of the MBS adducts on actin, we analyzed the tryptic peptide map of the modified protein using LC–ESI–MS/MS. The analysis was done separately for upper and lower bands of MB–actin excised from SDS gels.

Peptide mass mapping of the upper and lower bands covered 43 and 33% of actin sequence, respectively. To identify the peptides resolved by this method, observed molecular weights of the peptides were compared with the theoretical molecular weights of unmodified actin tryptic peptides generated in PeptideMass. The peptides that could not be ascribed to any theoretical peptide masses were identified by combining molecular weights of the theoretical peptides with the molecular weights of MBS adducts. The search for possible combinations of cross-linked peptides was restricted to the peptides containing Lys and Cys residues, which in the actin atomic structure are separated by approximately 6 to 11 Å (the length of a linker arm). The results of the search are summarized in Table 2 . The peptides found in the upper band are numbered from 1 to 5, and peptides found in the lower band are labeled 6 and 7.

### Characterization of MB–actin preparation based on chemical and MS methods

**Fig. 4:**
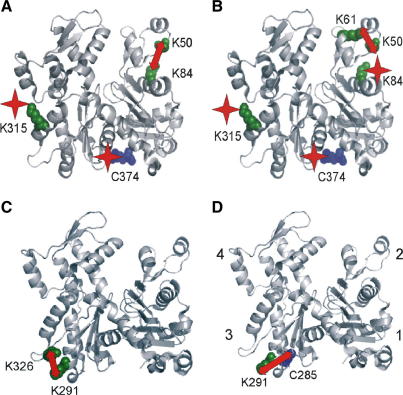
Actin sites modified with MBS. Lysine and cysteine residues involved in the reaction with MBS are highlighted in green and blue, respectively. The presence of a cross-link is marked by a red arrow, and labeling with MBS without cross-link formation is indicated by a red star. (A and B) Actin derivatives migrating in the upper band of SDS gels. (C and D) Modified actins migrating in the lower band of SDS gels. Actin subdomains 1 to 4 are indicated in panel D. Actin structure was plotted with the aid of PyMOL based on skeletal actin coordinates (PDB accession number 1j6z). (For interpretation of the references to color in this figure legend, the reader is referred to the Web version of this article.)

Taken together, the above results show that actin derivatives migrating in SDS gels in the upper and lower bands are combinations of differentially labeled molecules. The nanospray MS detected the presence of actin molecules that were either cross-linked (derivative I) or cross-linked and also labeled on one or two residues (derivatives II and III). The peptide mapping strategy shows that, indeed, such modifications exist. The modified sites localized in actin atomic structure are shown in Fig. 4 .

The molecules found in the upper band seem to be cross-linked between Lys50 and Lys84 and are also labeled on either Lys315 or Cys374 (derivative II) or on both Lys315 and Cys374 (derivative III) (Fig. 4A). The molecules with a cross-link between Lys50 and Lys61 can be labeled either on one of the residues Lys84, Lys315, or Cys374 (derivative II) or on two of the residues (derivative III) (Fig. 4B). Thus, the upper band is a mixture of actin molecules cross-linked in subdomain 2 with one or two MBS adducts attached to subdomains 1 and 3. Because these molecules do not separate on mini-gels, precise assessment of the populations of labeled molecules is beyond resolution of this method. However, intensity of the electrophoretic bands indicates that derivatives II and III consist of only 25% of the whole MB–actin preparation.

In the lower band, two types of cross-links were detected. Both contribute to the dominating derivative I (∼75% of the MB–actin preparation) and are localized in subdomain 3. It seems that the MB molecule initially attached to Lys291 is flexible enough to react with either Cys285 or Lys326 (Figs. 4C and D). Considering the fact that maleimide is more selective for Cys than for Lys, the cross-link with Cys285 is probably dominating; however, quantitative analysis of the ratio of both derivatives is not possible at this stage of investigations.

### Binding of tropomyosin isoforms to MBS- and FITC-modified actin

**Fig. 5:**
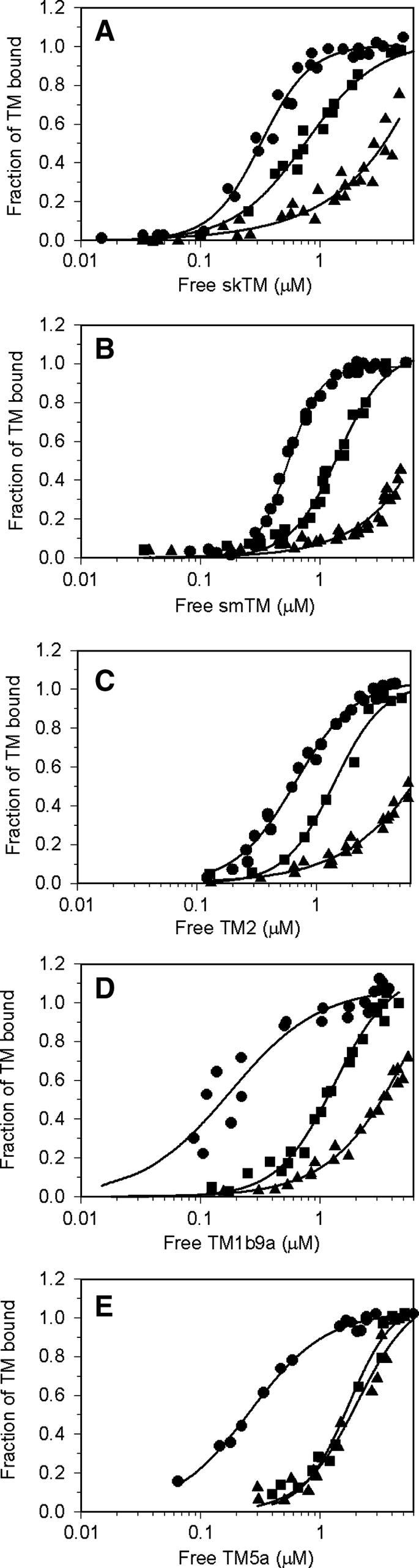
Effect of actin modification with MBS and FITC on its affinity for tropomyosin isoforms. Binding of the skTM (A), smTM (B), TM2 (C), TM1b9a (D), and TM5a (E) to native actin (circles), FITC–actin (squares), and MB–actin (triangles) in 5 mM imidazole (pH 7.0), 2 mM MgCl_2_, and 100 mM NaCl (or 30 mM NaCl in panel E) was analyzed as described in Materials and Methods. Symbols show collected experimental points from two to five experiments. Binding curves were generated by SigmaPlot by fitting the experimental points to a Hill equation (Eq. (1)).

The effect of actin modification with MBS on TM binding was tested with five α-tropomyosin isoforms: skTM, smTM, nonmuscle TM2, TM5a, and chimeric TM1b9a. The isoforms share similar sequence except for three regions encoded by alternative exons as described in Materials and Methods. Binding of TM isoforms to native F-actin and MB–F-actin, measured in a direct cosedimentation assay, is illustrated in Fig. 5 . The apparent equilibrium binding constants (*K*_app_), shown in Table 3 , indicate that actin modification reduced binding of all studied TM isoforms by more than an order of magnitude except for TM5a, which binds to MB–actin with an affinity approximately eightfold weaker than to native actin.

The results suggest that binding of TM isoforms is sensitive to actin conformation changed by the modification with MBS. Because in our MB–actin preparation the majority of actin molecules are cross-linked in subdomain 3, one can anticipate that subdomain 3 strongly contributed to tropomyosin binding, although binding of TM5a was affected to a smaller extent.

Gel electrophoresis and MS data showed that approximately 25% of MBS-modified actins carry adducts in subdomains 1 and 2. Unfortunately, we were unable to separate MB–actin cross-linked in subdomain 3 from the rest of labeled actins. We showed in our previous work that involvement of actin subdomain 1 in TM binding is small given that proteolytic removal of three C-terminal amino acids reduced affinity for TM isoforms no more than 1.5-fold [7,30]. Therefore, the major effect of actin labeling with MBS observed in this work must be due to subdomain 3 and subdomain 2 modifications.

To discern the contributions of both regions to cooperative actin binding, we tested TM binding to actin modified in subdomain 2 by covalent binding of FITC to Lys61 [20]. As shown in Fig. 5 and Table 3, affinities of FITC–actin for muscle TMs, TM2, and TM1b9a were intermediate between native actin and MB–actin. In contrast, the affinity constant of FITC–actin for TM5a was reduced eightfold and its value was close to the affinity of MB–actin.

### Cooperativity of TM isoforms binding to modified actin

According to the model developed by McGhee and von Hippel [31], two parameters contribute to the overall affinity (*K*_app_) of a long ligand binding to a linear lattice. The first parameter is an affinity of a ligand for an isolated site (*K*_0_), a site with no other ligands bound adjacently. The second parameter (ω) is a measure of binding cooperativity and describes an increase in affinity when a ligand’s binding is facilitated by near-neighbor interactions [4].

Using the McGhee–von Hippel equation, we analyzed the effects that actin modifications had on TM’s affinity for an isolated and contiguous site. In the case of MB–actin, the analysis was restricted to TM5a because we were not able to produce full binding curves for the other isoforms. The values of *K*_0_ and ω were obtained by fitting experimental points (shown in Fig. 5) to the McGhee–von Hippel equation (Eq. (2)). The data in Table 3 show that the parameters of the overall affinity reduction associated with actin modifications differed for each of the TM isoforms tested. The strongest effect of actin modifications on binding to an isolated site was observed for TM5a. In the case of other isoforms, *K*_0_ was less affected or unchanged (smTM). The cooperativity of TM binding was also differentially affected. Both actin modifications increased cooperativity of TM5a binding to a contiguous site. In contrast, other isoforms tended to bind to FITC actin with lower cooperativity than to native actin; however, the calculated difference in ω was not statistically significant for TM2 and TM1b9a.

## Discussion

Eukaryotic cells express multiple TM isoforms that often localize in different cellular compartments. The mechanism that drives the isoforms to different cellular regions is not known. One possible explanation is that binding of different TM isoforms is sensitive to actin conformation that may be determined by cellular location and the presence of other actin-binding proteins [3]. To analyze the mechanism of the thin filament assembly with various TM isoforms, we used actin in which the conformation was changed by covalent modifications with MBS and FITC.

Using chemical determinations and MS methods, we found that MBS modified actin by cross-linking Lys and Cys residues or binding to a single Lys in actin subdomain 3. Another cross-link was formed within subdomain 2 and/or between subdomains 2 and 1. Subdomains 2 and 1 can also be modified by attachment of a single label.

The precise location of fluorescein moiety of FITC attached to Lys61 is not known. However, analysis of the molecular distances between the Lys61 ε-amine group and actin amino acid residues within the radius of approximately 11 Å (a distance covered by FITC molecule) suggests that within an actin monomer the sphere of FITC influence potentially extends over a large part of subdomain 2.

Modifications with FITC and MBS gradually reduced the affinity of four muscle and nonmuscle TM isoforms toward actin. This observation suggests that actin subdomain 2 is important for binding of these isoforms, but the most important site of interactions is located in subdomain 3. In contrast, both modifications had similar effects on actin binding to nonmuscle TM5a. The latter observation was striking because MBS modifies actin much more extensively than does FITC. The data indicate that subdomain 2 strongly contributes to actin interactions with TM5a; however, this does not rule out involvement of subdomain 3 in binding this isoform. In the preparation of MB–actin, the molecules cross-linked in subdomain 3 are predominant species. If subdomain 3 were not involved in actin interactions with TM5a, MB–actin would bind to this tropomyosin with a higher affinity than FITC–actin, which is labeled on subdomain 2 only. This suggests that Tm5a binds to a discrete actin site that is similarly distorted by attachment of either FITC or MBS.

Our results also show that the factor that determines specificity of TM’s interactions with actin is not the length of TM but rather its sequence. Although TM5a and TM1b9a belong to the LMW class of isoforms, they were differentially affected by both actin modifications. Composition of the end-to-end overlap complex seems to be important for positioning of TM on actin. This conclusion is in line with our previous data obtained for C-terminally truncated actin [7].

One can speculate that modification with MBS had a significant effect on actin affinity for HMW TM isoforms and TM1b9a because these TMs bind close to the central part of subdomain 3. This subdomain was predicted as a major binding region of the skTM by an atomic model of actin–TM complex obtained by X-ray fiber diffraction [32]. The results obtained for yeast actin mutated in subdomain 3 support this notion [9]. However, if we consider our results in terms of TM orientation on the filament, it is hard to reconcile them with the three-dimensional reconstructions of electron microscopy images of actin filaments complexed with various TM isoforms. The reconstructions revealed that whereas skTM made contacts with subdomain 3 in the inner domain of actin filament, smTM was shifted toward the outer domain where interactions with subdomain 2 were more plausible. TM5a was found at a position indistinguishable from the location of skTM [33]. Thus, if TM5a binds to the same actin site as skTM, both isoforms should display similar sensitivity to actin modifications used in this work. The reason for differential binding of the isoforms to actin modified with MBS and FITC intrigues us. Our analysis suggests not only that direct actin–TM contacts are responsible for this phenomenon but also that allosteric changes within actin participate in TM isoform recognition.

The analysis of actin-binding data according to the McGhee–von Hippel model revealed that actin modifications differentially affected binding cooperativity and the affinity for an isolated site of each TM isoform. These results support our recent observation on actin modified by proteolytic removal of three C-terminal residues that actin truncation affected TM binding parameters in an isoform-dependent manner [7]. Because the end-to-end contacts between TMs were unaltered, the changes in *K*_0_ and ω observed in our experiments must be due to conformational changes in actin caused by the modifications. Therefore, the cooperativity coefficient must reflect TM-induced long-range interactions that are spread along an actin linear lattice. The idea that actin actively participates in the cooperativity of the thin filament has been proposed previously [34,35]. The findings of this and previous work [7] show that actin regions participating in cooperativity are spread throughout the entire molecule.

Taken together, our results support the idea that TM and actin are active partners responsible for functional differences of thin filaments working in various cellular environments.

## Figures and Tables

**Table 1:** Determination of free amine and thiol groups in native actin and MB–actin.

	Native actin	MB–actin
Free Cys374 SH group	0.95 ± 0.01	0.22 ± 0.03
Total reactive SH groups	4.91 ± 0.04	3.16 ± 0.05
Total reactive NH_2_ groups	15.40 ± 0.45	11.27 ± 0.26

*Note.* The reactive groups (mol/mol of actin) were determined in the fully folded proteins (Cys374) and also in unfolded proteins (total reactive groups). Averaged values from five or six experiments ± standard errors are shown. Conditions were as described in Materials and Methods.

**Table 2:** Modified peptides identified by ESI–LC–MS/MS.

	Observed mass [M+H]^+^	Calculated Mass (Da)	Position in sequence	Peptide sequence	Modification sites
1	2601.5	2600.2	40–50	HQGVM^a^VGM^a^GQK	K50–K61
			51–61	DSYVGDEAQSK	
2	3395.2	3392.6	40–50	HQGVM^a^VGM^a^GQK	K50–K84
			69–84	YPIEH^b^GIITNWDDM^a^EK	
3	2188.9	2191	69–84	YPIEH^b^GIITNWDDMEK	K84
4	639.1	639.4	313–315	M^a^QK	K315
5	482.2	484.1	374–375	CF	C374
6	1505	1506.9	291	K	K291–K326
			316–326	EITALAPSTMK	
7	1079.7	1079.6	291	K	K291–C285
			285–290	CDIDIR	

a Sulfoxide derivative.

b Methylated.

**Table 3:** Parameters of TM isoforms binding to native actin and modified actin.

TM isoform	Native F-actin			MB–F-actin			FITC–F-actin		
	*K*_app_ (μM^−1^)	*K*_0_ (μM^−1^)	ω	*K*_app_ (μM^−1^)	*K*_0_ (μM^−1^)	ω	*K*_app_ (μM^−1^)	*K*_0_ (μM^−1^)	ω
skTM	3.11 ± 0.13	0.127 ± 0.030	21.8 ± 3.3	<0.1	n.d.	n.d.	1.30 ± 0.08	0.082 ± 0.015	16.3 ± 2.9
smTM	1.84 ± 0.03	0.035 ± 0.006	38.7 ± 5.0	<0.1	n.d.	n.d.	0.70 ± 0.03	0.034 ± 0.007	22.3 ± 4.1
TM2	1.50 ± 0.06	0.075 ± 0.009	19.3 ± 2.1	<0.1	n.d.	n.d.	0.71 ± 0.04	0.039 ± 0.003	17.9 ± 1.4
TM1b9a	5.64 ± 0.91	0.367 ± 0.180	16.6 ± 6.2	<0.1	n.d.	n.d.	0.73 ± 0.07	0.065 ± 0.020	15.3 ± 4.4
TM5a	3.87 ± 0.17	0.377 ± 0.094	10.2 ± 2.7	0.49 ± 0.06	0.034 ± 0.005	16.9 ± 2.6	0.58 ± 0.09	0.035 ± 0.008	18.8 ± 3.6

*Note.* The apparent equilibrium binding constant (*K*_app_) was obtained by fitting the experimental points shown in Fig. 5 to the Hill equation (Eq. (1)). The affinity for an isolated site (*K*_0_) and the equilibrium constant for moving TM from an isolated binding site to a singly contiguous binding site (ω) were obtained by fitting the experimental data to the McGhee–von Hippel equation (Eq. (2)). Conditions: 5 mM imidazole (pH 7.0), 0.1 M NaCl (or 30 mM NaCl for TM5a), and 2 mM MgCl_2_. All parameters shown are averages ± standard errors. n.d., parameters not determined.

## Footnotes

1 *Abbreviations used:* TM, tropomyosin; HMW, high-molecular-weight; LMW, low-molecular-weight; MBS, *m*-maleimidobenzoyl-*N*-hydroxysuccinimide ester; MB–actin, maleimidobenzoyl-modified actin; MS, mass spectrometry; FITC, fluorescein-5-isothiocyanate; FITC–actin, actin derivative modified in subdomain 2 by attachment of FITC to Lys61; ATP, adenosine triphosphate; smTM, smooth muscle TM; skTM, skeletal muscle αTM; DMF, dimethyl formamide; SDS–PAGE, sodium dodecyl sulfate–polyacrylamide gel electrophoresis; DTNB, 5,5′-dithio-bis(2-nitrobenzoic acid); TNBS, 2,4,6-trinitrobenzenesulfonic acid; MS/MS, tandem MS; LC, liquid chromatography; ESI, electrospray ionization; DTE, dithioerythritol.
